# Autophagy is the main driver of radioresistance of HNSCC cells in mild hypoxia

**DOI:** 10.1111/jcmm.18482

**Published:** 2024-06-20

**Authors:** Rhianna M. Hill, Chun Li, Jonathan R. Hughes, Sonia Rocha, Gabrielle J. Grundy, Jason L. Parsons

**Affiliations:** ^1^ Institute of Systems, Molecular and Integrative Biology University of Liverpool Liverpool UK; ^2^ Institute of Cancer and Genomic Sciences University of Birmingham Birmingham UK

**Keywords:** autophagy, DNA damage repair, head and neck cancer, hypoxia, radiotherapy

## Abstract

Hypoxia poses a significant challenge to the effectiveness of radiotherapy in head and neck squamous cell carcinoma (HNSCC) patients, and it is imperative to discover novel approaches to overcome this. In this study, we investigated the underlying mechanisms contributing to x‐ray radioresistance in HPV‐negative HNSCC cells under mild hypoxic conditions (1% oxygen) and explored the potential for autophagy modulation as a promising therapeutic strategy. Our findings show that HNSCC cells exposed to mild hypoxic conditions exhibit increased radioresistance, which is largely mediated by the hypoxia‐inducible factor (HIF) pathway. We demonstrate that siRNA knockdown of HIF‐1α and HIF‐1β leads to increased radiosensitivity in HNSCC cells under hypoxia. Hypoxia‐induced radioresistance was not attributed to differences in DNA double strand break repair kinetics, as these remain largely unchanged under normoxic and hypoxic conditions. Rather, we identify autophagy as a critical protective mechanism in HNSCC cells following irradiation under mild hypoxia conditions. Targeting key autophagy genes, such as BECLIN1 and BNIP3/3L, using siRNA sensitizes these cells to irradiation. Whilst autophagy's role in hypoxic radioresistance remains controversial, this study highlights the importance of autophagy modulation as a potential therapeutic approach to enhance the effectiveness of radiotherapy in HNSCC.

## 1 INTRODUCTION

Head and neck squamous cell carcinoma (HNSCC) is the sixth most common cancer type worldwide and primarily begins in the squamous cells that line the mucosal surfaces of pharynx, larynx and oral cavity.^1^ Globally the cases of HNSCC are rising, with current incidences of ~800,000 cases/year predicted to increase by 34% by 2030.^2^ Risk factors for HNSCC include smoking, excessive alcohol consumption and human papillomavirus (HPV) infection.^3^, ^4^ Treatment for HNSCC invariably utilizes radiotherapy (ionising radiation; IR) which can be coupled with surgery and/or chemotherapy. IR kills tumour cells by generating DNA damage principally through the production of reactive oxygen species,^5^ and the formation of DNA double strand breaks (DSB) and complex DNA damage (containing two or more lesions within close proximity) are considered the most lethal type of DNA lesions causing the therapeutic effect.^6^ Interestingly, HPV‐positive cases of HNSCC are well established to have a better prognosis compared to HPV‐negative disease due to a better response to radiotherapy and chemotherapy as a result of deficiencies in DSB repair.^7^, ^8^, ^9^ Nevertheless, and upon induction of DNA damage, the DNA damage response is activated through a protein kinase signalling cascade that repairs the damage whilst also activating cell cycle arrest.^10^ DSBs are predominantly repaired via the non‐homologous end joining (NHEJ) pathway^11^ with homologous recombination (HR) being limited to the S/G2 phases of the cell cycle,^12^ whereas repair of CDD is likely to involve multiple pathways and enzymes.^6^ The formation of irreparable and persistent DNA damage can trigger activation of cell death pathways, including apoptosis, necrosis or autophagy.^13^

Despite radiotherapy being a key treatment modality for HNSCC, radioresistance still remains a problem leading to poor patient outcomes. One of the major factors contributing to radioresistance is tumour hypoxia. In the 1990s it was reported that HNSCC patients who had hypoxic tumours had a significantly reduced disease‐free survival following radiotherapy compared to patients with less hypoxic tumours.^14^, ^15^ It was subsequently observed that HNSCC patients with a low pre‐treatment oxygenation status within their tumour had worse loco‐regional tumour control compared to well oxygenated tumours.^16^ The oxygen fixation hypothesis (OFH) is used to explain how hypoxia generates radioresistance, whereby under these conditions this reduces the amount of fixed DNA damage making it easier for the cell to repair the damage.^17^ However, attempts to overcome the physical lack of oxygen have shown to be unsuccessful, and therefore, it is argued that the OFH does not fully explain the radioresistance mechanism particularly under mild hypoxia, and that other intrinsic cellular changes including effects on cell survival/death pathways contribute to this.

Hypoxia inducible factor (HIF) is a transcription factor which is stabilized under hypoxic conditions and helps cells to adapt and survive under such conditions.^18^ HIF consists of an oxygen regulated α‐subunit (HIF‐1α, HIF‐2α and HIF‐3α) and a β‐subunit (HIF‐1β) also known as aryl hydrocarbon nuclear translocator (ARNT),^19^, ^20^ and HIF‐1 upregulation is associated with pro‐tumorigenic effects through coordinating metabolic reprogramming, cellular proliferation, angiogenesis and modulating cell death mechanisms.^21^ Indeed in HNSCC, an overexpression of HIF has been associated with increased mortality.^22^ Furthermore, HIF has been proposed to contribute to hypoxia‐induced radioresistance and in a variety of different cancers inhibiting HIF has shown promising results in improving the radiation response.^23^, ^24^, ^25^, ^26^, ^27^ Specifically in patient derived primary oral squamous cell carcinoma cells, a negative correlation between radiosensitivity to γ‐rays and HIF‐1α is evident, and siRNA knockdown of HIF‐1α led to cellular radiosensitisation.^28^ Nonetheless, despite the well‐known and characterized significance of HIF in driving radioresistance in hypoxia, a considerable gap remains. Indeed, there is a lack of research comprehending the intricate molecular mechanisms that underlie how HIF induces hypoxic cellular radioresistance under mild conditions, and in HNSCC specifically. It is therefore important to investigate and identify these radioresistance and pro‐survival mechanisms further, which will aid the development of novel therapeutic targets and drugs to overcome hypoxic radioresistance in HNSCC patients.

In this study, we have analysed the comparative responses of HPV‐negative HNSCC cells grown in mild hypoxia (1% oxygen) versus normoxia to x‐ray irradiation, with a view to identifying the cellular mechanisms of radioresistance. This level of oxygen is also referred to as pathological hypoxia that disrupts normal homeostasis, which contrasts to radiobiological hypoxia involving much more severe oxygen levels (<0.4%).^29^ This oxygen concentration was chosen to reflect the relatively higher end of average oxygen tensions of tumours observed in HNSCC patients of 1.6–1.9% compared to physoxia (5% oxygen).^29^ We show that cells acquire a radioresistance phenotype in mild hypoxia, which to some extent is dependent on HIF expression, but not due to changes to DNA damage and repair. Instead, we reveal that cells modulate autophagy as a pro‐survival mechanism, and that targeting key genes within this pathway can lead to enhanced cellular radiosensitivity in hypoxia. This highlights the potential for targeting autophagy to overcome hypoxic radioresistance in HNSCC tumours leading to more effective treatment.

## 2 MATERIALS AND METHODS

### 2.1 Cell culture and siRNA knockdowns

HPV‐negative HNSCC cell lines UMSCC6, UMSCC74A and UMSCC12 (all originating from the larynx) were kindly provided by Prof. T. Carey (University of Michigan, USA). The FaDu cell line (originating from the hypopharynx) was acquired from ATCC (Teddington, UK). Cells were authenticated by STR profiling and mycoplasma tested regularly. All cell lines, except for FaDu which was cultured in Minimal Essential Medium, were cultured as monolayers in Dulbecco's Modified Eagle Medium with supplementation of 10% foetal bovine serum, 1% penicillin–streptomycin, 1% L‐glutamine and 1% non‐essential amino acids. Cells were maintained at 5% CO_2_ and 37°C. Unless where specifically mentioned, hypoxia refers to 1% oxygen for 16 h prior to irradiation and which was performed using the INVIVO_2_ 400 hypoxia chamber (Baker Ruskinn, Bridgend, UK). For siRNA knockdowns, cells were cultured in 35 mm dishes for 24 h until 30%–50% confluent, and then incubated with 2.5 μL of Lipofectamine RNAiMAX transfection reagent (Life Technologies, Paisley, UK), in the presence of 40 nM of either HIF‐1α, HIF‐1β and Beclin 1 siRNA, or 4 nM of either BNIP3 and BNIP3L siRNA for 48 h. The following siRNA sequences were used: Qiagen all‐stars negative control siRNA (Qiagen, Southampton, UK) as the non‐targeting control (NTC); ON‐TARGET PLUS siRNA pools against HIF‐1α and HIF‐1β from Horizon Discovery (Cambridge, UK), BECLIN 1 siRNA (5′‐CAGUUUGGCACAAUCAAUA‐3′) from Eurogentec (Seraing, Belgium). siRNA for BNIP3 (5′‐GGAUUACUUCUGAGCUUGCAACATA‐3′, 3′‐CACCUAAUGAAGACUCGAACGUUGUAU‐5′; 5′AUAUUGGAAGGCGUCUGACAACCUC‐3′, 3′‐GAUAUAACCUUCCGCAGACUGUUGGAG‐5′; 5′‐CUAUAUUGGAAGGCGUCUGACAACC‐3′, 3′‐UAGAUAUAACCUUCCGCAGACUGUUGG‐5′) and siRNA for BNIP3L (5′‐AUCUAUAUUGGAAAGCGACUGAGCA‐3′, 3′‐CGUAGAUAUAACCUUUCGCUGACUCGU‐5′; 5′‐GGUAUUUUCUCCGCAGAAUUUCUGA‐3′, 3′‐CCCCAUAAAAGAGGCGUCUUAAAGACU‐5′; 5′‐GACUUCUUACAGCUAGUGCAUUGTG‐3′, 3′‐CACUGAAGAAUGUCGAUCACGUAACAC‐5′) were both from Integrated DNA Technologies (Iowa, USA). The NTC for the double stranded BNIP3 and BNIP3L siRNA was NC1 from Integrated DNA Technologies (Iowa, USA).

### 2.2 Cell extract preparation and immunoblotting

Whole cell extracts were prepared as previously described.^9^ Briefly, cell pellets were resuspended in one packed cell volume of buffer containing 10 mM Tris–HCL (pH 7.8), 200 mM KCl, 1 μg/mL of each protease inhibitor (pepstatin, aprotinin, chymostatin and leupeptin), 1 mM PMSF and 1 mM DTT. Then two packed cell volumes of buffer containing 10 mM Tris–HCl (pH 7.8), 600 mM KCl, 40% glycerol, 0.1 mM EDTA, 0.2% Nonidet P‐40, 1 μg/mL of each protease inhibitor (pepstatin, aprotinin, chymostatin and leupeptin), 1 mM PMSF and 1 mM DTT was added to the suspension and mixed by rotation for 30 min at 4°C. The cell lysate was centrifuged at 40,000 rpm for 20 min, the supernatant collected and stored at −80°C. Protein extracts (usually 40 μg) were separated by Tris‐Glycine SDS‐PAGE and transferred onto an Immobilon FL PVDF membrane (Millipore, Watford, UK). The membranes were blocked overnight with Odyssey blocking buffer (Li‐Cor Biosciences, Cambridge, UK) and incubated overnight with primary antibody on a rotating platform at 4°C. Membranes were then washed with PBS containing 0.1% Tween 20 and incubated with either Alexa Fluor 680 (Thermo Fisher Scientific, Massachusetts, USA) or IR Dye 800 (Li‐Cor Biosciences, Cambridge, UK) secondary antibodies for 1 h at room temperature. Membranes were further washed with PBS containing 0.1% Tween 20 before being visualized and quantified using the Odyssey image analysis system (Li‐Cor Biosciences, Cambridge, UK). Antibodies against HIF‐1α and HIF‐1β were obtained from BD Biosciences (Wokingham, UK); p62, BNIP3 and BNIP3L antibodies were obtained from Cell Signalling Technology (Leiden, The Netherlands); BECLIN1 and LC3B antibodies were obtained from Abcam (Cambridge, UK).

### 2.3 Clonogenic survival assays

Defined numbers of cells were plated in triplicate in 6‐well plates and left to adhere for ~6 h before being subjected to mild hypoxia or normoxia overnight. Increasing cell numbers were plated for increasing IR doses to account for plating efficiencies, and cells had not divided prior to irradiation. Cells were treated with single doses of x‐rays using the 150 MeV CellRad x‐ray irradiator (Faxitron Bioptics, Tucson, USA) under mild hypoxic or normoxic conditions using customized Perspex boxes (designed by Prof. Ester Hammond, University of Oxford, UK), which were routinely tested for air tightness, to maintain the respective conditions during irradiation. All cells were then transferred to normoxic conditions at 37°C, 5% CO_2_ for 7–10 days to promote colony growth (>50 cells), after which they were stained with 6% glutaraldehyde and 0.5% crystal violet for 1 h. The plates were washed and left to air dry before the colonies were counted using the Gelcount colony analyser (Oxford Optronics, Oxford, UK). Statistical analysis of clonogenic survival data was conducted using the CFAssay for R package,^30^ which uses the linear‐quadratic (LQ) model to compare different treatment responses across increasing radiation doses.

### 2.4 Neutral comet assays

Neutral comet assays were performed similar to that previously described.^9^ In brief, cells grown in 35 mm dishes were subjected to normoxic or hypoxic conditions overnight prior to x‐ray irradiation (4 Gy) and then left to enable for DNA repair in their respective conditions. Cells were then trypsinised and diluted to ~1 × 10^5^ cells/ml, 250 μL cell suspension was embedded in 1% low melting point agarose onto a precoated agarose slide and allowed to set on ice. The slides were placed in fresh cold lysis buffer (2.5 M NaCl, 100 mM EDTA disodium salt, 10 mM Tris base, 1% N‐lauroylsarcosine, 1% DMSO, and 1% (v/v) Triton X‐100; pH 9.5) for at least 1 h at 4°C, and then transferred to an electrophoresis tank and covered with fresh cold electrophoresis buffer (1× TBE; 90 mM Tris–borate, 2 mM EDTA, pH 8.3) for 30 min to allow DNA unwinding. Electrophoresis was performed at 25 V for 25 min, slides were removed and washed with 1× PBS three times for 5 min and then left to air dry overnight. Slides were rehydrated in water (pH 8) for 30 min and stained with SYBR Gold (Thermo Fisher Scientific, Massachusetts, USA) diluted 1:20,000 in water (pH 8.0) for 30 min. The slides were visualized using an Olympus fluorescent microscope with a Photometrics CoolSNAP HQ2 CCD camera, and images were captured using Micro‐Manager Software. The images were analysed using Komet 6.0 image analysis software (Andor Technology, Belfast, Northern Ireland) to determine % tail DNA values from at least three independent, biological experiments.

### 2.5 Immunofluorescence microscopy

Cells were grown on 13 mm glass coverslips, transferred to hypoxia or normoxia overnight, treated with x‐rays (4 Gy), and cells then left to enable for DNA repair in their respective conditions. Cells were then washed with PBS, fixed using 4% paraformaldehyde for 10 min, permeabilized with 0.2% Triton X‐100 in PBS for 10 min, then washed three times with 0.1% Tween‐20 for 10 min. The cells were blocked with 2% BSA for 30 min on a rocking platform at room temperature before incubation with antibodies against γH2AX (Abcam, Cambridge, UK) or 53BP1 (Bethyl Laboratories, Montgomery, USA) overnight at 4°C on a rocking platform. The coverslips were washed three times with 1× PBS before incubation for 1 h at room temperature on a rocking platform with Alexa Fluor 555‐conjugated secondary antibody (Thermo Fisher Scientific, Massachusetts, USA). The coverslips were then washed with 1× PBS for 10 min before being mounted with Fluoroshield containing DAPI (Merck Life Science, Gillingham, UK). Cells were examined using an Olympus BX61 fluorescent microscope with a Photometrics CoolSNAP HQ2 CCD camera, and MicroManager software was used to capture images. ~100–200 cells were analysed per experiments across 5–6 images, and at least three independent, biological experiments were performed.

### 2.6 Gene expression analysis

FaDu cells were seeded into 35 mm dishes and grown until they reached ~70% confluency, and then placed in either normoxia or hypoxia (1% oxygen for 16 h). Cells were unirradiated or irradiated using 4 Gy x‐rays using customized Perspex boxes to maintain the respective conditions during irradiation, and then allowed to recover for 4 or 24 h before being harvested by centrifugation at 1500 rpm for 5 min at 4°C. Sample processing and analysis was conducted by Dr Frances Greaney‐Davies and Dr Mike Davies at the University of Liverpool. The HTG EdgeSeq platform along with the HTG EdgeSeq Oncology Biomarker Panel of 2560 genes was used according to the manufacturer's instructions (HTG Molecular Diagnostics, USA), and gene expression data was analysed using the HTG Reveal software.

### 2.7 Statistical analysis

For clonogenic survival assays, statistical analysis was conducted from three independent biological experiments using the CFAssay statistical analysis package for R.^30^ This test uses the LQ model to compare different treatment responses across increasing radiation doses. For comparisons between DSB levels using the comet assay and γH2AX/53BP1 foci at individual time points post‐irradiation, Student's *t‐*tests were performed, analysed across three independent, biological repeats.

## 3 RESULTS

### 3.1 Mild hypoxia induces radioresistance in HPV‐negative HNSCC cells

**FIGURE 1:**
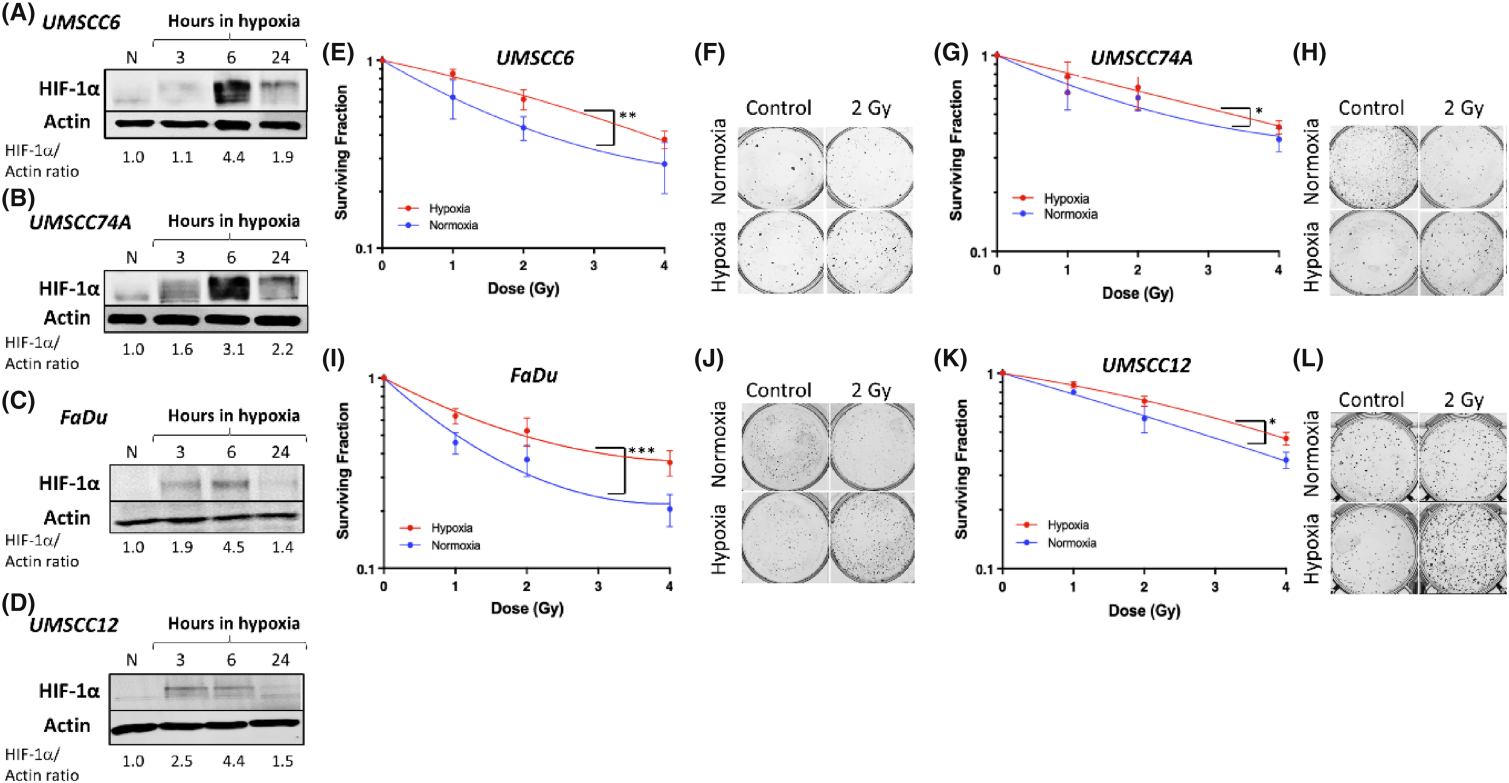
Mild hypoxia induces radioresistance in HPV‐negative HNSCC cells. (A) UMSCC6, (B) UMSCC74A, (C) FaDu and (D) UMSCC12 cells were exposed to normoxia (N) or mild hypoxic conditions (1% oxygen) for 3, 6 and 24 h, whole cell extracts were prepared and analysed by immunoblotting. Alternatively, clonogenic survival of (E,F) UMSCC6, (G,H) UMSCC74A, (I,J) FaDu and (K,L) UMSCC12 cells following normoxia and hypoxia (1% oxygen for 16 h) treatment prior to exposure to increasing doses of x‐ray radiation was analysed. (E, G, I and K) Shown is surviving fraction ± SE from three independent experiments with LQ fitting, as well as (F, H, J and L) representative images of the colonies formed from unirradiated and irradiated (2 Gy; where four times the numbers of cells were seeded) conditions. *p < 0.03, **p < 0.01, ***p < 0.001 as analysed by the CFAssay.

We initially analysed the induction of HIF‐1α, as a major driver of the cellular response in hypoxia, in four HPV‐negative HNSCC cell lines at different time points following incubation in mild hypoxic conditions (1% oxygen) which is the focus of this study. Under these conditions, we observed that HIF‐1α protein levels began to increase at 3 h and was largely maximal at 6 h in hypoxia in UMSCC6, UMSCC74A, FaDu and UMSCC12 cells (Figure 1A–D). As expected, a cyclical protein expression was observed in which HIF‐1α levels were reduced 24 h after hypoxic exposure. Using clonogenic assays, we then analysed the impact of mild hypoxia (1% oxygen for 16 h) on the survival of HPV‐negative HNSCC cell lines to x‐ray irradiation. Here we observed that all four cell lines in hypoxia demonstrated a statistically significant increase in radioresistance, compared to cells grown in normoxia (UMSCC6, *p* < 0.001; UMSCC74A, *p* < 0.03; FaDu, *p* < 0.01; UMSCC12, *p* < 0.03) (Figure 1E–L). Interestingly, we observed that cells appeared to display differential responses to mild and severe (0.1% oxygen) hypoxia, and where the latter enhanced radioresistance further in FaDu cells whereas there were no changes observed in UMSCC6 cells (Figure S1A,B). In contrast, and despite the hyperoxygenated conditions used as the normoxic control, we found no significant differences in survival post‐irradiation between HNSCC cells incubated in 20% (normoxia) versus 5% (physoxia) oxygen (Figure S1C,D).

**FIGURE 2:**
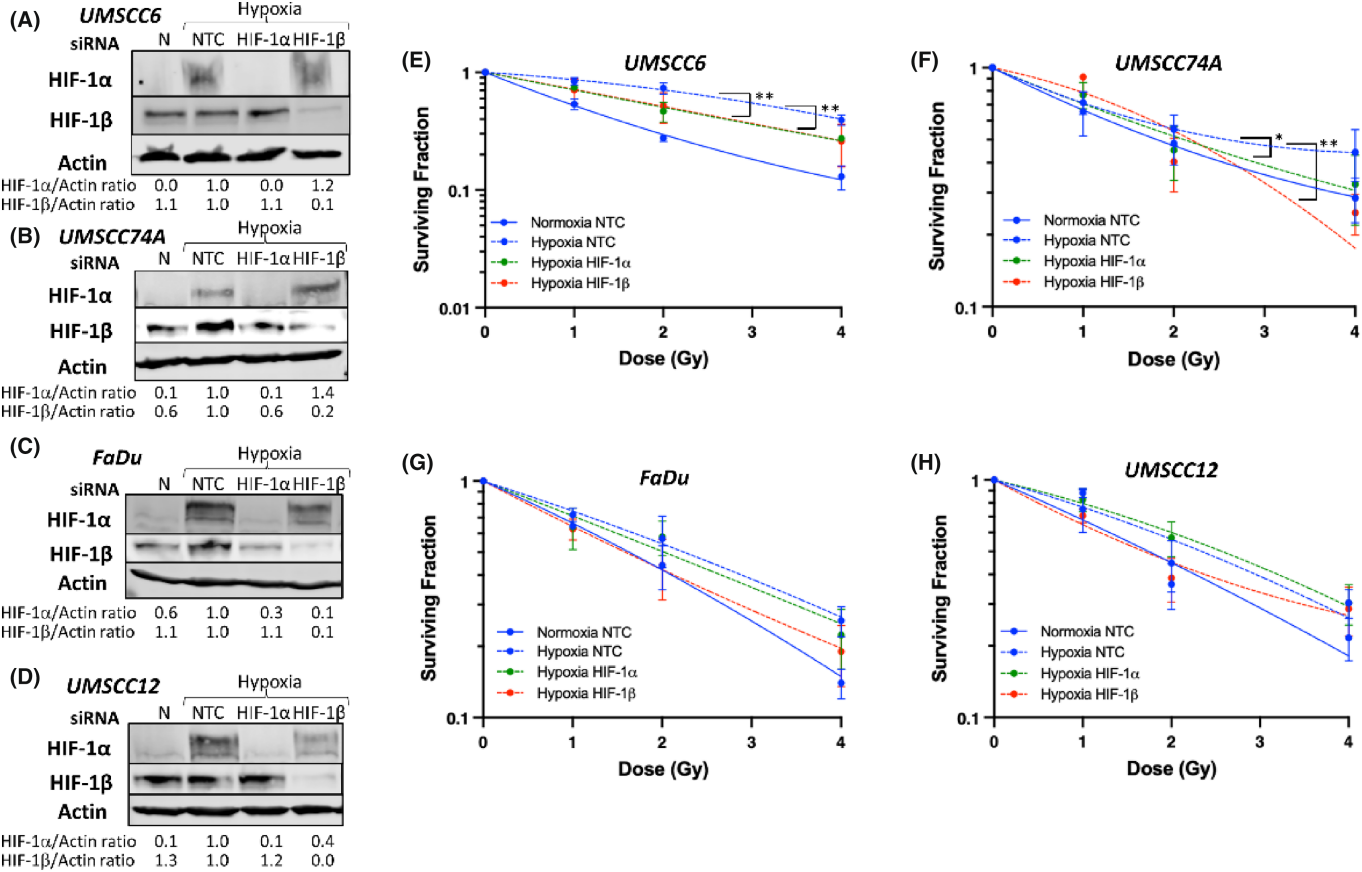
The radioresistance of HPV‐negative HNSCC cells in mild hypoxia shows some dependence on HIF1. (A) UMSCC6, (B) UMSCC74A, (C) FaDu and (D) UMSCC12 cells were treated with HIF‐1α or HIF‐1β siRNA, versus a non‐targeting control siRNA (NTC) for 48 h, followed by incubation in mild hypoxia (1% oxygen) for 6 h prior to preparation of whole cell extracts and analysis by immunoblotting. Extracts prepared from cells incubated in normoxia (N) as a control are also shown. Alternatively, clonogenic survival of (E) UMSCC6, (F) UMSCC74A, (G) FaDu and (H) UMSCC12 cells following an siRNA knockdown in normoxia or hypoxia (1% oxygen for 16 h) were analysed in response to increasing doses of x‐ray radiation. (E–H) Shown is surviving fraction ± SE from three independent experiments with LQ fitting. *p < 0.007, **p < 0.001 as analysed by the CFAssay.

We subsequently utilized siRNA knockdowns of HIF‐1α, but also HIF‐1β which would supress all HIF related activity, to assess the impact on hypoxia‐induced radioresistance. Immunoblotting analysis confirmed the successful siRNA knockdown efficiencies as observed by the >70% reduction in the protein levels of HIF‐1α or HIF‐1β with the respective siRNA treatments in all four HNSCC cell lines following exposure to hypoxia for 6 h (Figure 2A–D). Analysis of clonogenic survival revealed a statistically significant increase in the radiosensitivity of UMSCC6 and UMSCC74A cells with either a HIF‐1α siRNA knockdown (*p* < 0.001 and *p* < 0.007 respectively) or a HIF‐1β siRNA knockdown (*p* < 0.001 for both cell lines) under hypoxic conditions compared to the non‐targeting siRNA control (NTC) treated cells (Figure 2E,F). Interestingly in FaDu and UMSCC12 cells, an siRNA knockdown of HIF‐1α appeared to have a limited effect on cellular radiosensitivity under hypoxic conditions, whereas a HIF‐1β siRNA knockdown did appear to induce radiosensitisation compared to the NTC cells in hypoxia but which was not statistically significant (Figure 2G,H). It should be noted that the NTC control response curves in these experiments had a slightly reduced surviving fraction compared to those previously performed (Figure 1), most likely attributed to the transfection procedure and which includes the NTC siRNA.

### 3.2 DSB levels and repair are not associated with hypoxia‐induced radioresistance in HPV‐negative HNSCC cells

**FIGURE 3:**
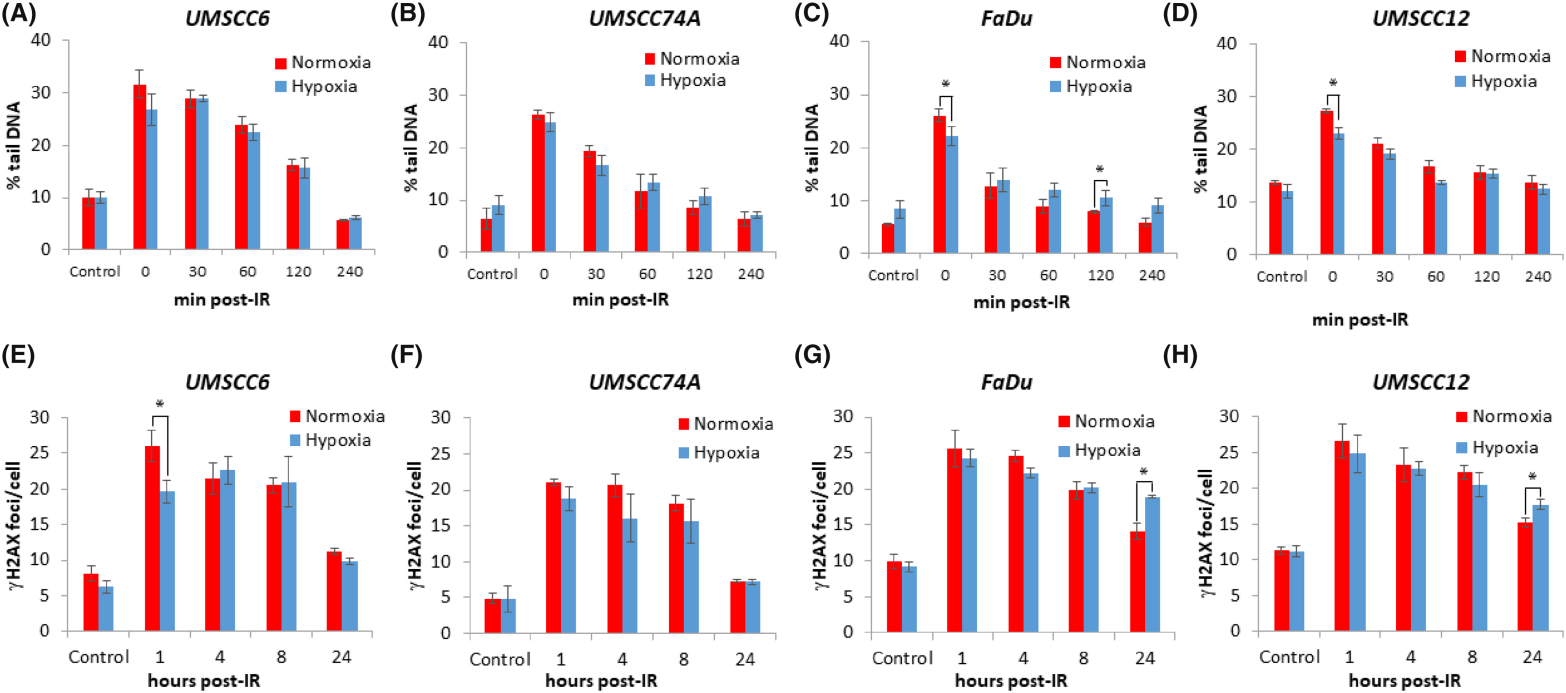
The radioresistance of HPV‐negative HNSCC cells in mild hypoxia is not driven through changes in DSB levels and their repair. (A, E) UMSCC6, (B, F) UMSCC74A, (C, G) FaDu and (D, H) UMSCC12 cells were exposed to normoxia or mild hypoxia (1% oxygen for 16 h) prior to x‐ray (4 Gy) irradiation. (A–D) DNA double strand breaks were analysed at various time points post‐irradiation by neutral comet assays, and shown is the mean % tail DNA ± SE from three independent experiments. (E–H) Alternatively, γH2AX foci were analysed using immunofluorescence microscopy and shown are the average numbers of γH2AX foci/cell from three independent experiments. *p < 0.05 as analysed by a two sample *t*‐test.

To examine whether radioresistance of HPV‐negative HNSCC cells in mild hypoxic could be explained by altered DNA DSB levels or repair kinetics, we utilized the neutral comet assay as well as γH2AX foci analysis as a surrogate DSB marker. In UMSCC6 and UMSCC74A cells, the levels of DSBs in unirradiated cells and in cells immediately post‐irradiation was not significantly different comparing normoxic versus hypoxic conditions (Figure 3A,B, see Control and time 0). Additionally, the kinetics of DSB repair post‐irradiation were the same under both normoxic and hypoxic conditions, and where the majority of DSBs were repaired between 120 and 240 min post‐irradiation (Figure 3A,B). Similar observations were seen in FaDu and UMSCC12 cells. Despite there being a statistically significant decrease in DSB levels immediately post‐irradiation in hypoxia versus normoxia in these cells, the gradual resolution of the damage over a period of 30–240 min was generally not significantly different comparing cells in normoxia versus hypoxia (Figure 3C,D; representative images for each cell line are displayed in Figure S2A–D). This evidence was confirmed on examination of γH2AX foci in cells pre‐ and post‐irradiation. In UMSCC6 cells, there was statistically significantly less γH2AX foci in hypoxic versus normoxic cells at 1 h post‐irradiation, although no statistically significant differences were observed at 4–24 h post‐irradiation. This illustrates similar DNA repair kinetics under the different conditions in these cells (Figure 3E). In UMSCC74A, FaDu and UMSCC12 cells, there was similarly no significant differences in the kinetics of resolving of γH2AX foci at 1–8 h post‐irradiation comparing normoxic and hypoxic conditions (Figure 3F–H; representative images for each cell line are displayed in Figure S3A–D). A statistically significant increase in γH2AX foci in FaDu and UMSCC12 cells at 24 h post‐irradiation was seen in hypoxic versus normoxic cells, although this would not correlate with increased cellular radioresistance under such conditions. Interestingly on examination of 53BP1 foci in UMSCC74A and FaDu cells as another marker of DSB repair, this revealed that the levels of 53BP1 foci were significantly lower at 1–4 h post‐irradiation in both cells in hypoxia versus normoxia (Figure S4A,B). 53BP1 foci levels were also significantly lower at 24 h post‐irradiation in FaDu cells in hypoxia. This suggests that the levels and/or recruitment of 53BP1 to DSB sites is lower in HNSCC cells in hypoxia, but which again would not support the relationship of this to increased radioresistance under these conditions. We also analysed the levels and activation of the key DSB protein kinases, ataxia telangiectasia mutated (ATM) and DNA‐dependent protein kinase (DNA‐PK) by immunoblotting in UMSCC74A and FaDu cells. However, and in line with the DSB repair data, we found no evidence to suggest that these were associated with radioresistance, and in fact the levels of these kinases were~30%–50% reduced under hypoxia versus normoxia (Figure S5A,B). Levels of 53BP1, but also the HR protein RAD51, were similarly reduced (by ~50%–60%) in hypoxia, suggesting a reduced capacity for performing DSB repair via HR under these conditions (Figure S5C,D). Nevertheless collectively, this suggests that there is no significant contribution of enhanced DNA DSB signalling and repair which would account for the increased radioresistance of HPV‐negative HNSCC cells in mild hypoxia.

### 3.3 Targeting autophagy can overcome the radioresistance of HPV‐negative HNSCC cells under hypoxia

**FIGURE 4:**
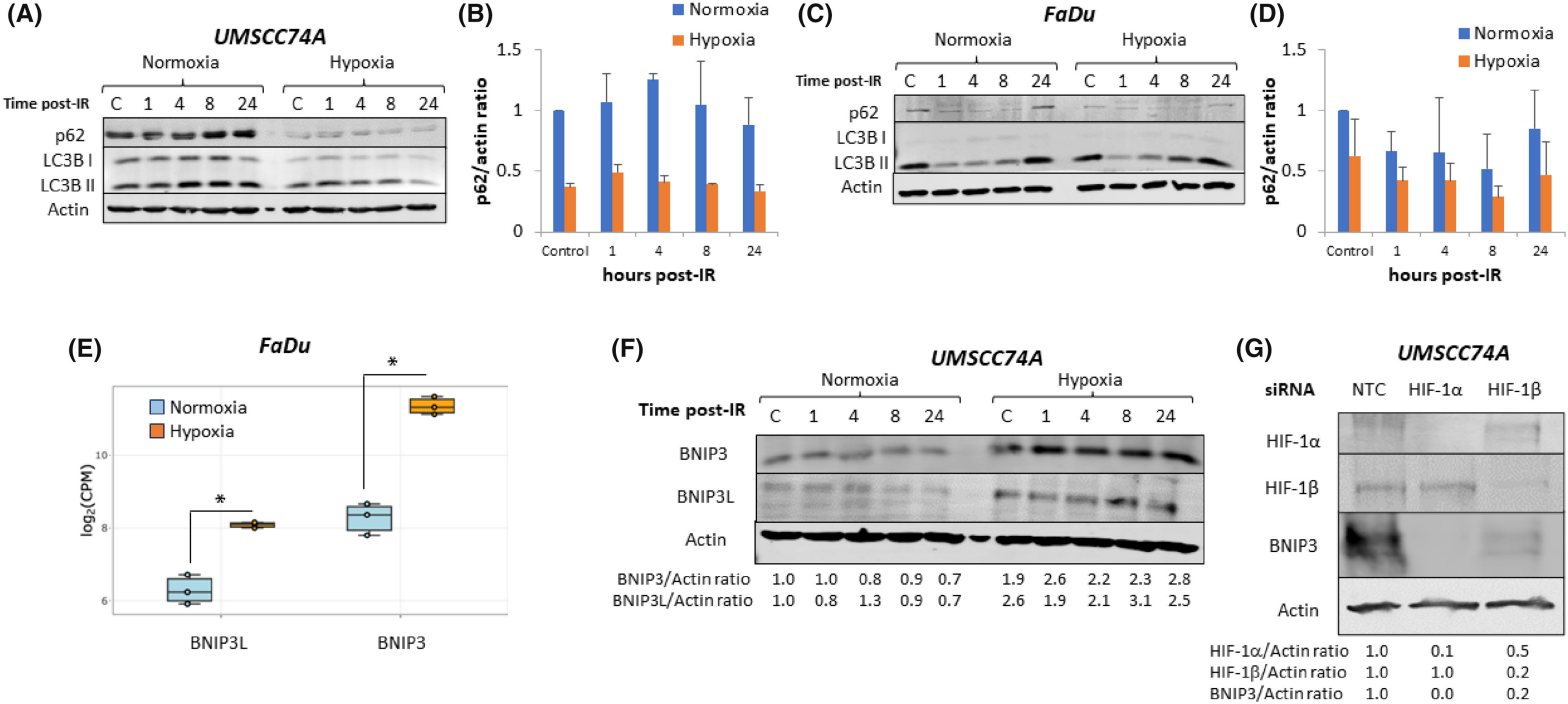
Autophagy is induced in HPV‐negative HNSCC cells in mild hypoxia. (A, B and F) UMSCC74A and (C, D) FaDu cells were exposed to normoxia or hypoxia (1% oxygen for 16 h) prior to x‐ray irradiation (10 Gy), whole cell extracts were prepared at the indicated times post‐irradiation and analysed by immunoblotting. (B, D) Protein levels of p62 normalized to the normoxic control were analysed from two independent experiments. (E) Gene expression analysis of BNIP3 and BNIP3L in FaDu cells exposed to either normoxia or hypoxia. *p < 0.001 as analysed by a two sample *t*‐test. (G) UMSCC74A cells were treated with HIF‐1α or HIF‐1β siRNA, versus a non‐targeting control siRNA (NTC) for 48 h, followed by incubation in mild hypoxia (1% oxygen) for 6 h prior to preparation of whole cell extracts and analysis by immunoblotting.

Autophagy has previously been shown to be an important pro‐survival mechanism under hypoxic conditions, therefore we decided to investigate the contribution of this cellular process to hypoxia‐induced radioresistance in HPV‐negative HNSCC cells. On immunoblotting analysis of the autophagy proteins p62 and LC3B in UMSCC74A cells, we observed a significant decrease in level of these proteins when cells were in hypoxia versus normoxia, and which was maintained at between 1 and 24 h post‐irradiation (Figure 4A,B). A decrease in p62 abundance suggests heightened autophagic activity, as p62 is degraded during this process. Similar observations were seen in FaDu cells (Figure 4C,D), although the magnitude of the difference in p62 and LC3B protein levels under the comparative conditions was less than that observed in UMSCC74A cells. Additionally, through gene expression analysis, we observed that the autophagy inducers BNIP3 and BNIP3L were upregulated in FaDu cells in mild hypoxia compared to normoxia, and which was confirmed at the protein level in UMSCC74A cells using immunoblotting (Figure 4E,F). The association between HIF‐1 and BNIP3 regulation is furthermore demonstrated by the substantial reduction in BNIP3 protein levels in UMSCC74A cells upon siRNA knockdown of both HIF‐1α and HIF‐1β (Figure 4G).

**FIGURE 5:**
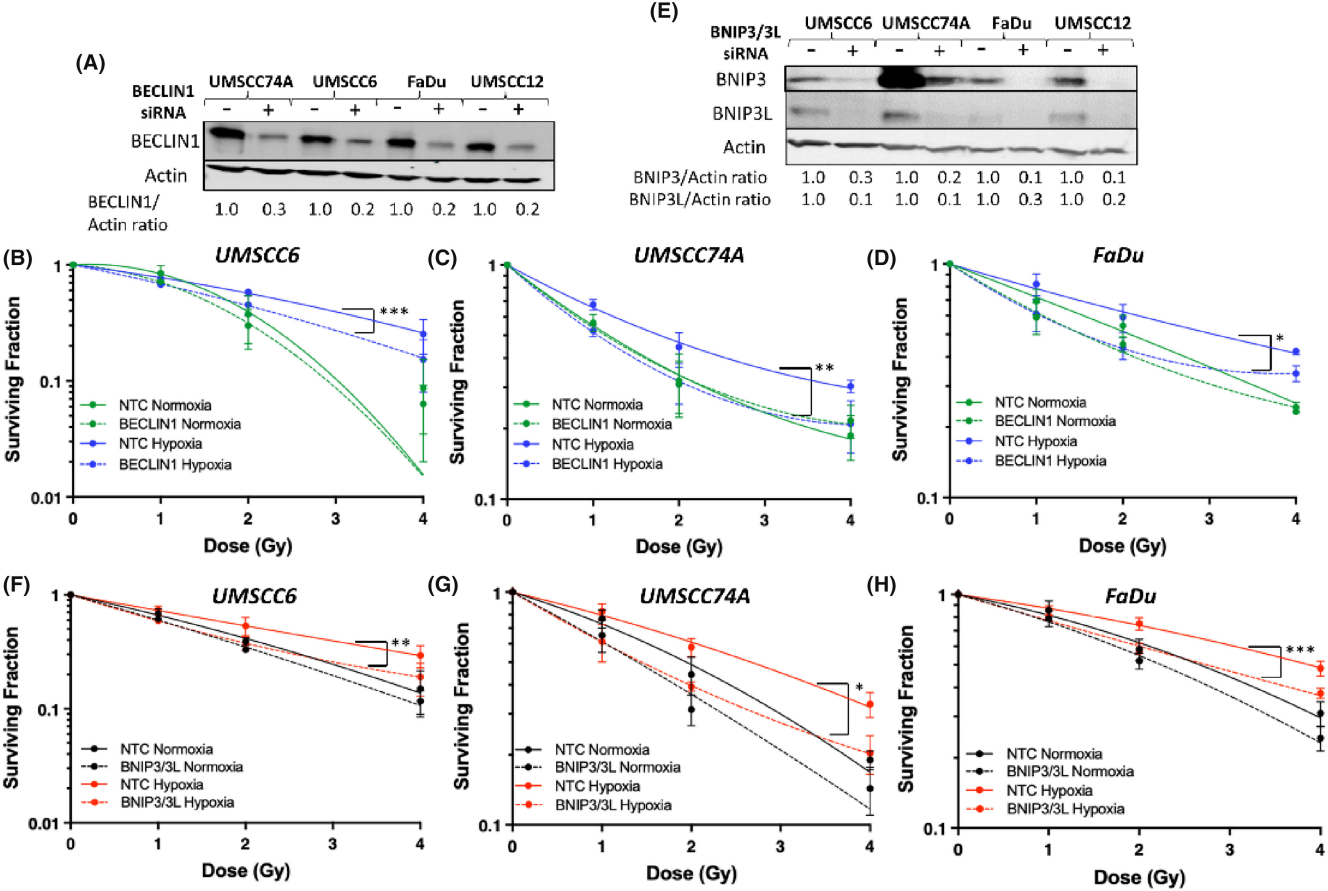
Autophagy induction plays a major role in the radioresistance of HPV‐negative HNSCC cells in mild hypoxia. UMSCC6, UMSCC74A and FaDu cells were treated with (A–D) BECLIN1 or (E‐H) BNIP3/3L siRNA, versus a non‐targeting control siRNA (NTC) for 48 h. (A and E) Whole cell extracts were prepared from cells and analysed by immunoblotting. (B–D and F–H) Clonogenic survival of UMSCC6, UMSCC74A and FaDu cells following an siRNA knockdown in normoxia or hypoxia (1% oxygen for 16 h) were analysed in response to increasing doses of x‐ray radiation. Shown is surviving fraction ± SE from three independent experiments with LQ fitting. *p < 0.05, **p < 0.03, ***p < 0.007 as analysed by the CFAssay.

To investigate the link between autophagy and radioresistance, we targeted BECLIN1 as a key regulator of autophagy using siRNA, which was shown to be >80% effective at the protein level in UMSCC6, UMSCC74A and FaDu cells (Figure 5A). Using clonogenic survival assays, we demonstrate that a BECLIN1 siRNA knockdown compared to the NT targeting control siRNA (NTC) caused a statistically significantly increase in radiosensitisation of UMSCC6 (*p* < 0.007), UMSCC74A (*p* < 0.03) and FaDu (*p* < 0.05) cells under hypoxic conditions (Figure 5B–D). Cell survival under these conditions were comparable to those incubated in normoxia using the NTC siRNA. No statistically significant difference in survival of the cells was observed under normoxic conditions in the absence of BECLIN1. We subsequently strengthened this evidence using a dual siRNA knockdown of BNIP3 and BNIP3L. Following a successful siRNA knockdown of BNIP3/3L (Figure 5E), we demonstrate that similar to targeting BECLIN1, an absence of BNIP3/3L causes a statistically significant increase in the radiosensitivity of UMSCC6 (*p* < 0.03), UMSCC74A (*p* < 0.05) and FaDu (*p* < 0.007) cells under hypoxic conditions (Figure 5F–H). Again, the cell survival profile under these conditions were similar to cells incubated in normoxia in the presence of a NTC siRNA. Interestingly, targeting BNIP3/3L also appeared to increase radiosensitivity of particularly UMSCC74A and FaDu cells in normoxia, although this was not statistically significant. Nevertheless, this furthers the idea that autophagy is one of the major drivers of hypoxia‐induced radioresistance in HPV‐negative HNSCC cells.

## 4 DISCUSSION

It is well known and established that hypoxia is a major barrier to radiotherapy success in HNSCC patients, although there has been little progress in developing novel therapeutics to overcome hypoxia and which can improve lead to improve patient outcomes and survival post‐radiotherapy.^31^ Here, we show evidence that HPV‐negative HNSCC cells grown in culture can gain a radioresistance phenotype in mild hypoxia versus normoxia, which appears HIF‐dependent. However, we find no apparent association of hypoxia‐induced radioresistance with an alteration in DSB signalling or processing, rather we provide evidence that autophagy is the major factor associated with this phenotype, and that targeting key autophagy genes can overcome radioresistance.

We confirmed in vitro that mild hypoxic conditions (1% oxygen for 16 h) significantly increased the radioresistance of HPV negative HNSCC cell lines, which has also been seen at more severe levels of hypoxia (<0.1%).^32^ However, the intrinsic molecular mechanisms driving these effects at the different oxygen tensions are expectedly different. Nevertheless, HIF‐1 is very much the central coordinator of the hypoxic response where it promotes the transcription of many genes essential for cellular survival. Previous work has identified HIF to be an important contributor to hypoxia‐induced radioresistance of different cancer cell lines,^23^, ^24^, ^25^, ^26^, ^28^ however there has been limited work in HNSCC. Our research utilising siRNA knockdown of HIF‐1α and HIF‐1β to demonstrate an increase in radiosensitivity of HNSCC cell lines under hypoxia, also support the key role of HIF in this process. Including the knockdown of HIF‐1β allows for the inhibition of activity of both HIF‐1 and HIF‐2 complexes. Importantly analysing the TCGA database, in HNSCC tumour tissue there is a significantly increased expression of HIF‐1α transcripts compared to normal tissue (Figure S6A). This difference is not shown for HIF‐2α suggesting that HIF‐1α may specifically play an important role in HNSCC, and which likely drives radioresistance. There is some evidence to suggest that inhibiting HIFs function may not improve radiosensitivity under hypoxia.^27^, ^33^ In colorectal cancer (HCT116) cells subjected to 0.5% oxygen then reoxygenated for 10 min prior to irradiation, it was found that HIF‐1 may actually act as a radiosensitiser through coordinating apoptosis and proliferation rates, but also that the impact of HIF‐1 inhibition on radiosensitivity is likely to be dependent on the physiological microenvironment.^27^ In terms of the molecular mechanism driving radioresistance, we discovered that DSB levels and repair post‐irradiation were similar between HNSCC cells irradiated under mild hypoxia and normoxia. This correlates with another study in HNSCC cell lines exposed to mild oxygen following photon irradiation which showed little differences in γH2AX foci in normoxia versus hypoxia.^34^ However, most evidence investigating hypoxia and relationship with DNA damage repair has been conducted in severe levels of hypoxia (<0.1% oxygen) which makes comparisons difficult since under these conditions many DNA repair genes are repressed leading to residual γH2AX post‐irradiation that subsequently promotes genomic instability.^35^, ^36^ However interestingly, it has been shown that when non‐small cell lung carcinoma cell lines were exposed to x‐ray irradiation under severe hypoxia, most of the DNA damage was effectively repaired yet DNA repair gene expression was repressed.^37^ This confirms the complexity of events occurring under both mild and severe hypoxic conditions.

In contrast to DNA repair, we observed that autophagy is activated in HPV‐negative HNSCC cells in mild hypoxia observed through decreases in p62, and increases in BNIP3 and BNIP3L gene and protein levels. The magnitude of the differences in autophagy activation in hypoxia between the cell lines, as revealed by p62 protein levels in UMSCC74A versus FaDu cells, appears to be different. Therefore, further exploration of the factors driving this response are needed in a broader panel of well characterized HNSCC cell lines. According to the TCGA database, BNIP3, BNIP3L and BECLIN1 gene expression levels are also elevated in HNSCC compared to normal tissue, and high expression of BNIP3 and BECLIN1 is associated with decreased patient survival (Figure S6A,B). Additionally, we observed that an siRNA knockdown of HIF‐1α and HIF‐1β reduced the protein levels of BNIP3, demonstrating the important interaction between HIF and autophagy regulation Furthermore, we also found that autophagy acts as a protective mechanism in HPV‐negative HNSCC cells post‐irradiation under mild hypoxia, and where targeting BECLIN1 or BNIP3/3L using siRNA can radiosensitize the cells to x‐rays. Autophagy is an evolutionary preserved process which helps to maintain cellular homeostasis, however more recently it has also been considered a cell death mechanism. Autophagy has been shown to be upregulated under hypoxic conditions, including at 1% oxygen, in a variety of different cancer cell lines,^38^, ^39^, ^40^ and has been linked with increasing cellular survival.^41^ The activation of autophagy under hypoxia has been linked with HIF‐1α‐dependent upregulation of key autophagy proteins, such as BNIP3 and BECLIN1,^41^, ^42^, ^43^ which correlates with our data. However, the role that autophagy plays in response to ionising radiation is controversial, with evidence for a protective role but also a cell death mechanism,^44^, ^45^, ^46^, ^47^, ^48^ and which likely reflects tumour specificity. Similar to our observations, siRNA‐mediated knockdown of BECLIN1 was found to radiosensitise breast cancer cells in hypoxia due to a delay in DSB repair.^49^ Furthermore, the use of chloroquine as an autophagy inhibitor, has been shown to radiosensitise lung and osteosarcoma cancer cell lines under hypoxia.^42^, ^50^ HIF‐1α has been linked with playing an important role in promoting hypoxia‐induced autophagy in cancer cells through its targeting of BNIP3/3L.^51^, ^52^, ^53^ In colon cancer cells, it has been observed that HIF‐1α‐induced autophagy increased cellular radioresistance,^54^ but also that HIF‐1α siRNA increased the radiosensitivity of breast cancer cells due to a reduction in autophagy.^55^ Our data now expands on this evidence by demonstrating that HNSCC cells also enhance the expression of autophagy‐related genes and proteins in mild hypoxia leading to a radioresistance phenotype.

In summary, our study elucidates an important role for autophagy and its subsequent modulation in the context of hypoxic radiotherapy in HPV‐negative HNSCC cells. We believe this highlights a potential avenue for therapeutic intervention whereby targeting key autophagy genes can help improve HNSCC cancer treatment. However, further research using more advanced preclinical models, including those in mice, are necessary to explore this to its full potential.

## AUTHOR CONTRIBUTIONS

**Rhianna M. Hill:** Data curation (lead); investigation (lead); methodology (equal); writing – original draft (lead). **Chun Li:** Data curation (equal); investigation (equal). **Jonathan R. Hughes:** Data curation (equal); investigation (equal). **Sonia Rocha:** Supervision (equal); writing – review and editing (equal). **Gabrielle J. Grundy:** Supervision (equal); writing – review and editing (equal). **Jason L. Parsons:** Conceptualization (lead); funding acquisition (lead); methodology (equal); project administration (lead); supervision (equal); writing – review and editing (equal).

## CONFLICT OF INTEREST STATEMENT

The authors declare no potential conflicts of interest.

## Supporting information


Figure S1.

## Data Availability

The authors declare that all data supporting the findings of this study are available within the article and its supplementary information, or from the corresponding author on reasonable request.
