# Supplementary material for: Autophagy is the main driver of radioresistance of HNSCC cells in mild hypoxia
Source: J Cell Mol Med. 2024 Jun 20;28(12):e18482. doi: 10.1111/jcmm.18482 (PMC11187736; doi:10.1111/jcmm.18482)
Supplement: Supplementary file 1 — Figure S1. [file JCMM-28-e18482-s001.docx]

**Autophagy is the main driver of radioresistance of HNSCC cells in mild hypoxia**

Rhianna M. Hill^1^, Chun Li^2^, Jonathan R. Hughes^2^, Sonia Rocha^1^, Gabrielle J. Grundy^1^, Jason L. Parsons^2,*^

^1^Institute of Systems, Molecular and Integrative Biology, University of Liverpool, Liverpool, L7 8TX, UK

^2^Institute of Cancer and Genomic Sciences, University of Birmingham, Edgbaston, Birmingham, B15 2TT, UK

*To whom correspondence should be addressed:

Tel: (44) 121 414 6850; email: [j.parsons.3@bham.ac.uk](mailto:j.parsons@liverpool.ac.uk)

**Supplementary data**

**
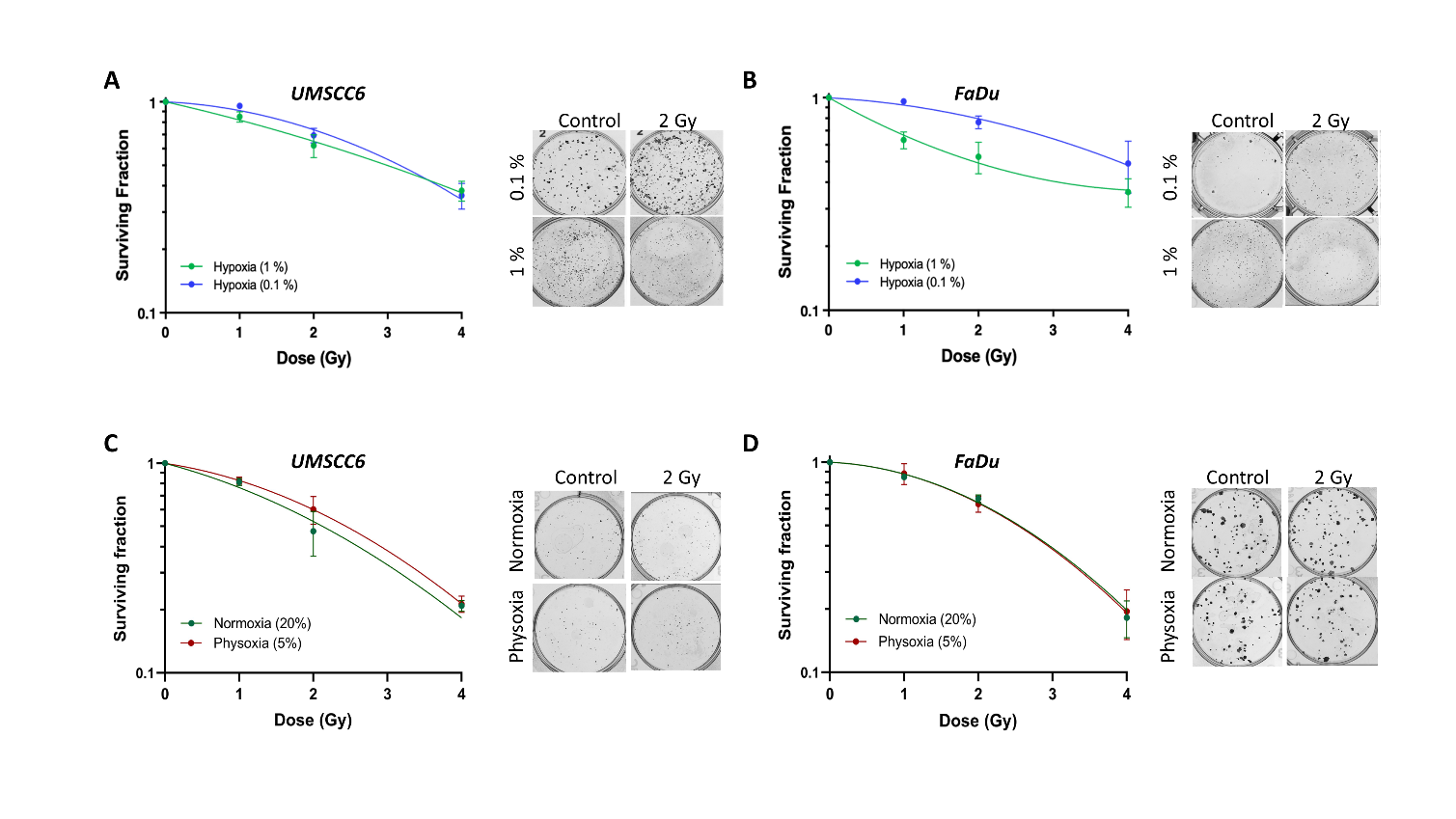
**

**Supplementary Fig 1.** Comparative survival of HPV-negative HNSCC cells post-irradiation in different oxygen conditions. (**A** and **C**) UMSCC6 and (**B** and **D**) FaDu cells were exposed to (**A-B**) severe or mild hypoxic conditions (0.1 % or 1 % oxygen), or (**C-D**) normoxia or physoxia (20 % or 5 % oxygen) for 16 h prior to treatment with increasing doses of X-ray radiation. Clonogenic survival was analysed, and data are shown as surviving fraction±SE from three independent experiments along with representative images of the colonies formed from unirradiated and irradiated (2 Gy; where four times the numbers of cells were seeded) conditions.


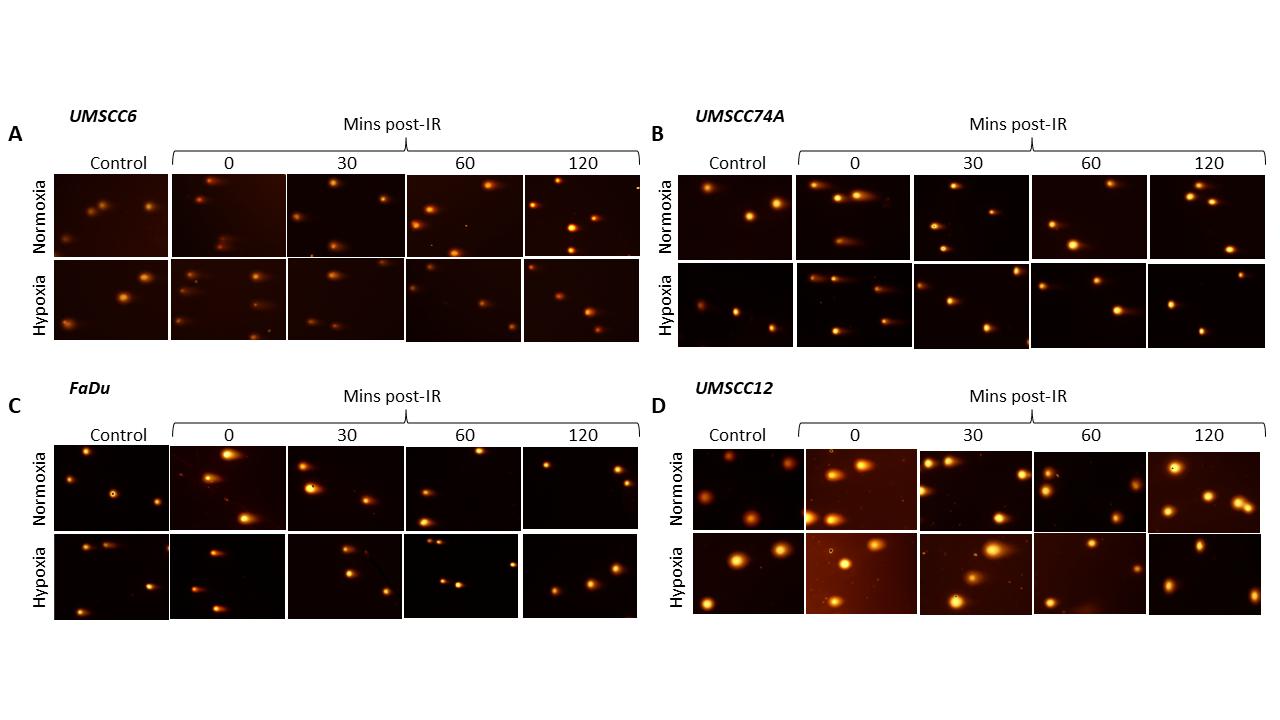


**Supplementary Fig 2**. Comet assay images demonstrating the levels and repair of X-ray-induced DNA double strand breaks in HPV-negative HNSCC cells in mild hypoxia versus normoxia. (**A**) UMSCC6, (**B**) UMSCC74A, (**C**) FaDu and (**D**) UMSCC12 cells were exposed to normoxia or mild hypoxia (1 % oxygen for 16 h) prior to X-ray (4 Gy) irradiation. DNA double strand breaks were analysed at various time points post-irradiation by neutral comet assays, and where respective images are shown.


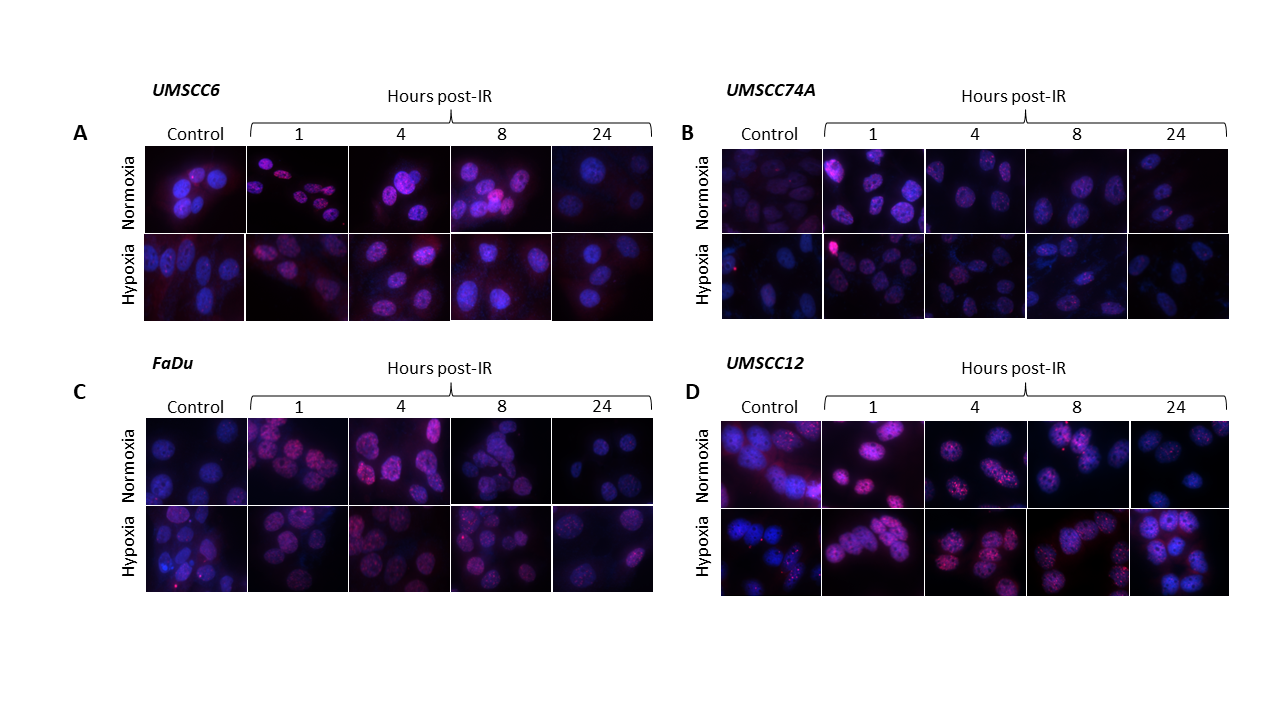


**Supplementary Fig 3**. Images of γH2AX foci in HPV-negative HNSCC cells in mild hypoxia versus normoxia following X-ray radiation. (**A**) UMSCC6, (**B**) UMSCC74A, (**C**) FaDu and (**D**) UMSCC12 cells were exposed to normoxia or mild hypoxia (1 % oxygen for 16 h) prior to X-ray (4 Gy) irradiation. γH2AX foci were analysed at various time points post-irradiation using immunofluorescence microscopy, and respective images are shown.

**
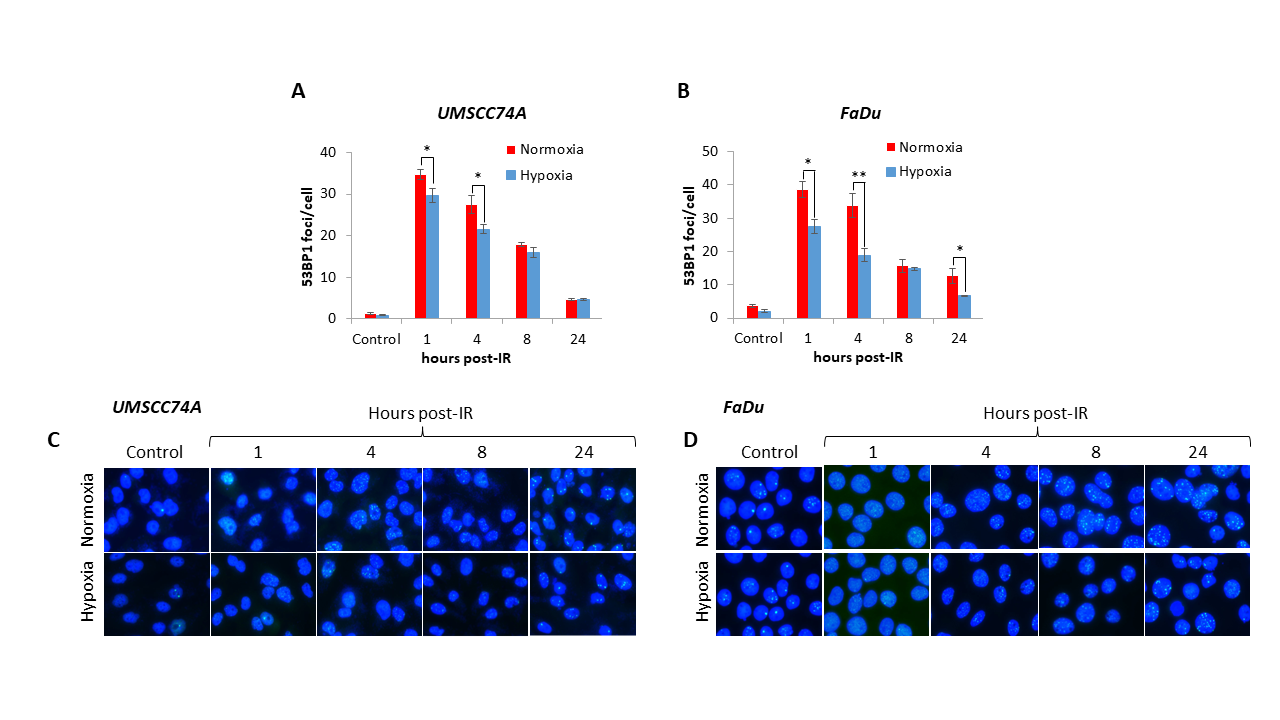
**

**Supplementary Fig 4**. Analysis of 53BP1 foci in HPV-negative HNSCC cells in mild hypoxia versus normoxia following X-ray radiation. (**A**) UMSCC74A and (**B**) FaDu were exposed to normoxia or mild hypoxia (1 % oxygen for 16 h) prior to X-ray (4 Gy) irradiation. 53BP1 foci were analysed at various time points post-irradiation using immunofluorescence microscopy. (**C** and **D**) Respective images of 53BP1 foci are shown.


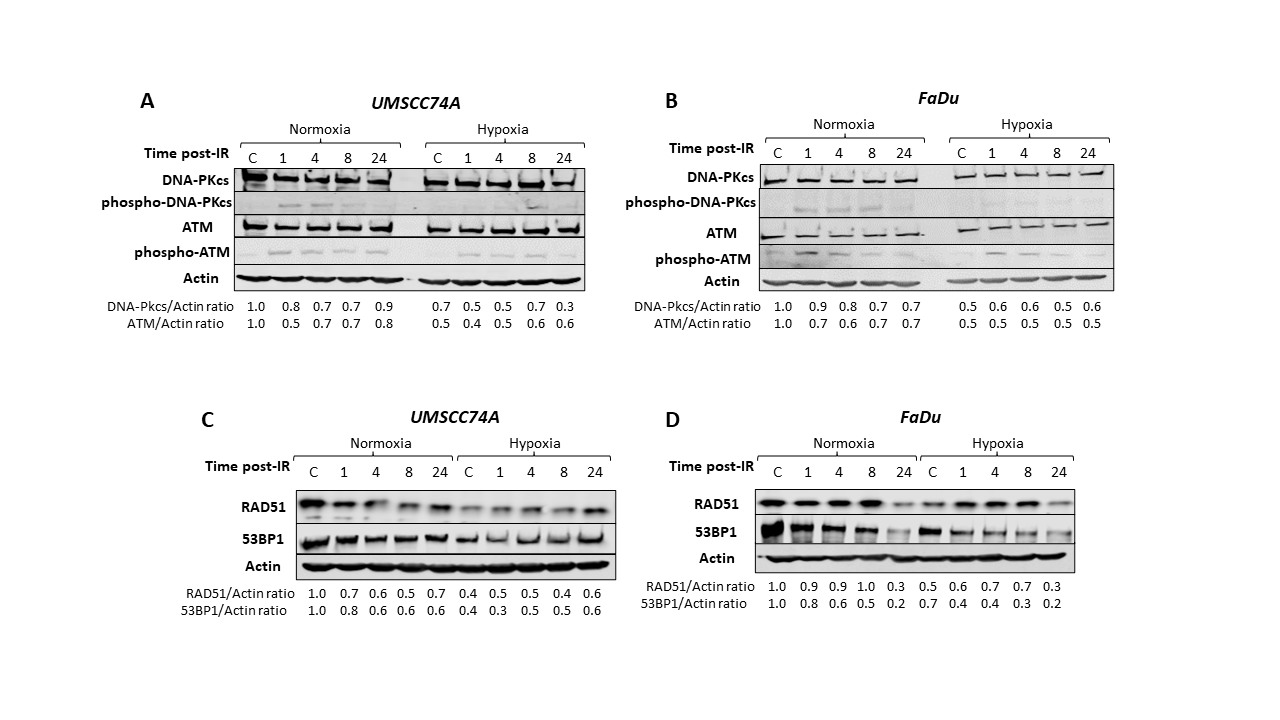


**Supplementary Fig. 5** Analysis of the levels of DNA double strand break repair proteins in HNSCC cells in hypoxia and normoxia in response to X-ray irradiation. (**A** and **C**) UMSCC74A or (**B** and **D**) FaDu cells were exposed to normoxia or mild hypoxic conditions (1 % oxygen for 16 h) prior to X-ray (4 Gy) irradiation, and cells harvested at the time points indicated. Whole cell extracts were prepared and analysed by immunoblotting with the indicated antibodies.


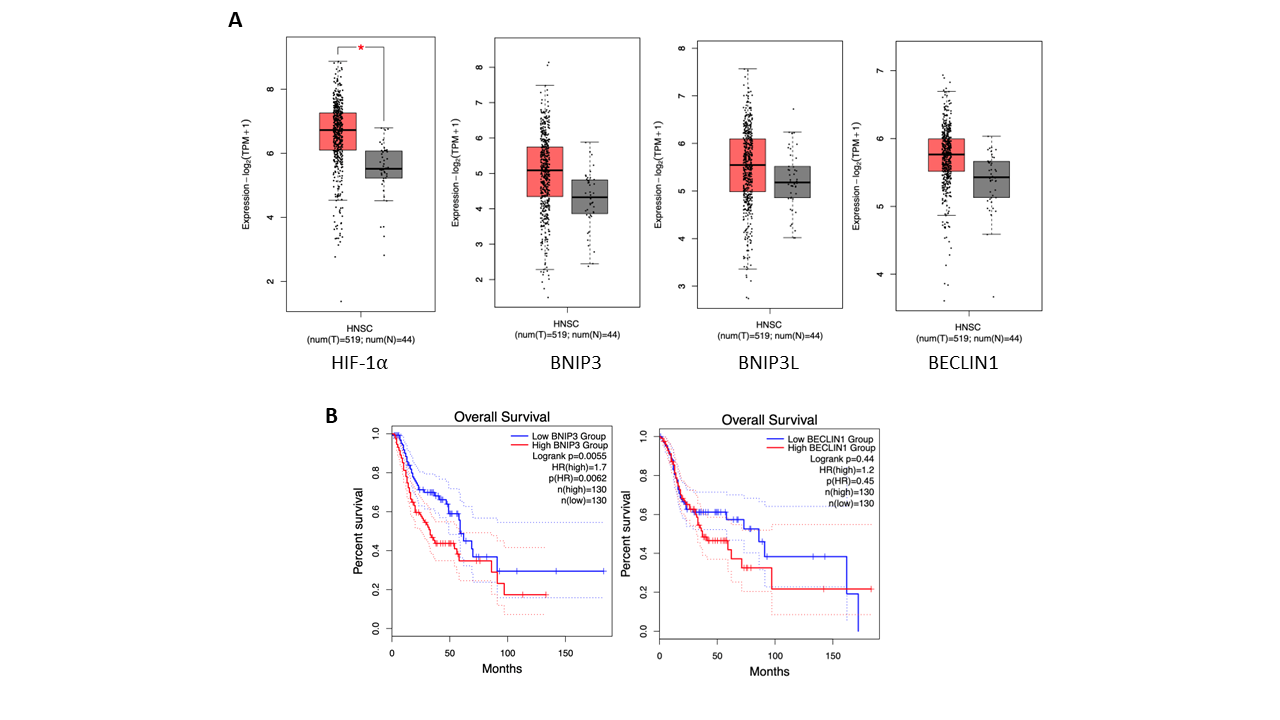


**Supplementary Fig 6**. Analysis of the expression levels of key autophagy genes in HNSCC patient samples and the relationship to overall survival. (**A**) Analysis of the expression levels of HIF1α, BNIP3, BNIP3L and BECLIN1 in HNSCC versus normal tissue acquired form the TCGA database. *p<0.05 as analysed by a student’s *t*-test. (**B**) Correlation of high expression levels of BNIP3 and BECLIN1 with reduced overall survival of HNSCC patients.
